# Virtual Rejection and Overinclusion in Eating Disorders: An Experimental Investigation of the Impact on Emotions, Stress Perception, and Food Attitudes

**DOI:** 10.3390/nu15041021

**Published:** 2023-02-17

**Authors:** Paolo Meneguzzo, Valentina Meregalli, Enrico Collantoni, Valentina Cardi, Elena Tenconi, Angela Favaro

**Affiliations:** 1 Department of Neuroscience, University of Padova, Via Giustiniani 2, 35128 Padova, Italy; 2 Neuroscience Padova Center, University of Padova, Via Orus 2, 35128 Padova, Italy; 3 Department of Psychological Medicine, Institute of Psychiatry, Psychology and Neuroscience, King’s College London, London WC2R 2LS, UK; 4 Department of General Psychology, University of Padova, Via Venezia 8, 35128 Padova, Italy

**Keywords:** anorexia nervosa, bulimia nervosa, binge eating disorder, Cyberball, ostracism, overinclusion, food attitudes

## Abstract

(1) Background: the investigation of how interpersonal functioning affects eating psychopathology has been receiving increasing attention in the last decade. This study evaluates the impact of virtual social inclusion or ostracism on emotions, perceived stress, eating psychopathology, and the drive to binge or restrict in patients across the eating disorder spectrum. (2) Methods: a group of 122 adolescent and adult females with different eating disorder diagnoses were compared to 50 healthy peers with regards to their performance on, and responses to the Cyberball task, a virtual ball-tossing game. Each participant was randomly assigned to playing a social inclusion or a social exclusion block of the Cyberball task and completed self-report assessments of emotions, perceived stress and urge to restrict/binge before and after the task. (3) Results: patients with anorexia nervosa showed a more negative impact on psychological well-being evaluated with the need threat scale after the excluding block, while patients with bulimia nervosa reported more negative effects after the overincluding condition. Patients with binge eating disorder showed a reduction in specific negative emotions after the overincluding block, unlike all other participants. (4) Conclusions: findings show significant correlations between restraint thoughts in patients with bulimia nervosa and binge thoughts in patients with binge eating disorder after being exposed to the inclusion condition. Different reactions in cognitive and emotional states of patients with eating disorders after different interpersonal scenarios confirm the impact of inclusive or exclusive relationships on eating psychopathology, with specific and different responses across the eating disorder spectrum, that have been discussed, linked to their eating behavioral cognition.

## 1. Introduction

Despite differences in their clinical manifestations, people with eating disorders (EDs) often experience interpersonal problems and difficulties with social cognition that negatively affect their social functioning, quality of life, and daily activities [1,2,3,4,5]. Different psychological elements have been related to dysfunctional eating behaviors and interpersonal difficulties [6]. Individuals with EDs have generally reported the presence of maladaptive coping strategies in various social contexts, showing dysfunctional emotional responses that are modulated through eating disorder symptoms, such as caloric restriction, bingeing, purging, or compulsive exercise [6,7,8]. For example, the fear of negative evaluation and social anxiety are vulnerabilities in individuals with EDs whose symptomatology includes body dissatisfaction, bulimic symptoms, shape concerns, weight concerns, and eating concerns [9,10]. Instead, less is known about the fear of positive evaluation in EDs, even if it is a relevant factor in social anxiety [11,12]. Indeed, the fear of others’ positive judgments has been positively correlated to high levels of social and meal-related anxiety, with possible connections to others’ expectation, clinical perfectionism, and concerns about mistakes [9,13]. These psychological aspects have been related to dysfunctional behaviors in EDs and are predictors of outcomes [14]. Looking at the general population, fear of positive evaluation has been positively related to bulimic symptoms, body dissatisfaction, weight, and shape concerns [9]. However, data are still preliminary, and no structured study is available about this specific social features in these clinical populations.

Looking at the cognitive aspects of emotional functioning, patients with anorexia nervosa (AN) demonstrate reduced emotional expression, difficulties understanding their own and others’ emotional and mental states, submissive attitudes and behaviors, and social inhibition in their relationships [15,16,17,18]. Similarly, patients with bulimia nervosa (BN) and binge eating disorder (BED) are struggling with identifying their feelings and managing interpersonal relationships [19]. Although more evidence is needed, these findings suggest a possible impairment in social skills across the entire ED spectrum [3,20,21,22,23]. 

The literature has shown that greater sensitivity to social rejection may lead to social difficulties, along with a more negative interpretation of ambiguous social situations [24]. Since the perception of being excluded or ignored by others represents a potential threat to the fundamental psychological need to be accepted, it is usually experienced as a painful situation and could have negative consequences, including the exacerbation of ED symptoms [25]. Some studies on healthy individuals have shown that being ostracized impairs participants’ self-regulation and increases their weight concerns and motivation to eat [26,27]. Looking at clinical samples, one study [28] observed that patients with BN reported an increase in hunger and desire to binge after completing an imagery task designed to elicit feelings of lack of appreciation and rejection. Only a few studies, however, have examined how patients with EDs interpret and react to social interactions, largely focusing on social exclusion [6], with no study available on the effects of social overinclusion. The fear of positive evaluations has been related to eating psychopathology as negative judgments [9,13], but actual overinclusion may be a different construct—and it has been neglected in the literature. Positive evaluations may trigger similar psychopathological responses as negative ones because individuals may interpret them as equally judgmental [29,30]. Yet, the previous literature—perhaps because patients tend toward social isolation—has neglected this aspect of eating psychopathology, and comparisons between social exclusion and inclusion are needed.

One task commonly used to assess participants’ reactions to interpersonal dynamics is the Cyberball task [31], a computerized ball-tossing game that can manipulate the degree of social inclusion by varying the number of times a participant is passed the ball. This task has been used to assess exclusion and overinclusion in both healthy individuals and patients with neurodevelopmental and psychiatric conditions such as autism spectrum disorder, major depression, social anxiety, and particularly in borderline personality disorder [32,33,34]. The Cyberball task has also been applied in studies of interpersonal relationships and eating behaviors to show the effects of social exclusion on food assumptions [35,36,37]. The Cyberball conditions of exclusion and inclusion/overinclusion have been related several times, respectively, to the fear of negative and positive evaluations [38,39,40], but studies specifically addressing the ED population are lacking. In a previous study, we implemented the Cyberball task in a sample of patients with AN using exclusion and overinclusion paradigms; our results showed that although patients with AN tended to overestimate their actual exclusion and displayed higher sensitivity to exclusion than their peers, they had no self-reported changes in the mood [41]. Similarly, in the overincluding task, they did not report any difference compared to the controls, corroborating the idea that fear of positive evaluation may be more linked to food anxiety and other social aspects [13], but studies on the population with bulimic symptoms are needed. 

Despite the finding that patients with AN experience greater sensitivity to exclusion than healthy peers, it is largely unknown whether people with a different ED diagnosis (i.e., BN or BED) would respond similarly or whether virtually induced social exclusion or overinclusion have a different impact on their emotions, stress levels, and eating attitudes. Thus, the present study seeks to extend previous findings among patients with AN by including participants with BN or BED in our assessment of the impact of virtual exclusion and overinclusion, looking at mood and eating behaviors. Our first aim was to evaluate the effects of exclusion and overinclusion in patients with an ED compared to community peers. We hypothesized that patients with different EDs would present different emotional and cognitive responses to exclusion and overinclusion. Individuals with AN may confirm their lack of response to exclusion, while individuals with BN or BED might be more sensitive to overinclusion, due to the preliminary data in the general population [9]. Our second aim was to evaluate the effects of interpersonal interactions on emotions, stress, and attitudes toward food, examining specific differences between participants who might have relevant clinical effects. Regarding clinical features, more restrictive responses were expected in patients with AN, while more eating-mediated responses were expected in patients with binge episodes. Finally, an exploration of the specific correlations between changes in emotions and attitudes toward food would help clinicians to understand any links between eating behaviors and interpersonal difficulties.

## 2. Materials and Methods

### 2.1. Participants

Participants were recruited from the University Hospital of Padova (Italy) at the local Eating Disorder Center. A group of 122 patients (AN (*n* = 42), BN (*n* = 40), and BED (*n* = 40)) was included. For comparison, a group of 50 healthy women (HW) was also recruited from the general population through non-specific public announcements seeking volunteers and during meetings in public high schools and at the University of Padova. Parts of the AN (30 out of 42) and HW (34 out of 50) groups were also included in our previous paper [41]. The inclusion criteria for both groups were as follows: female gender; between 15 and 40 years old; no severe psychiatric (e.g., bipolar disorder or schizophrenia) or medical comorbidity (e.g., severe hypotension, bradycardia, or electrolyte alterations), neurological trauma, or disorder. Additional criteria for the HW group were no lifetime diagnosis of ED, anxiety, or mood disorders. Inclusion and exclusion criteria were evaluated via a clinical interview performed by a trained psychiatrist, and the ED diagnoses agreed with Diagnostic and Statistical Manual of Mental Disorders (DSM-5) criteria [42]. Before participating in the study, each participant (and parents of participants younger than 18 years of age) was given written consent. All participants were volunteers and did not receive any credits or payments. The local ethics committee approved the study as part of a more extensive study on the cognitive evaluation of ED patients, and the study complies with the provisions of the Declaration of Helsinki.

### 2.2. Materials

Demographic and clinical data (height, weight, age at menarche, education, medications) were directly detected by researchers during the assessment of the participants. This included the duration of the disorder for ED patients, measured by the time (in months) from the beginning of ED symptomatology to the moment of the study.

#### 2.2.1. Self-Reported Measures

The psychological assessment was composed of various self-report questionnaires. The Eating Disorders Examination Questionnaire (EDE-Q), a 28-item questionnaire, assesses eating psychopathology and eating disorder behaviors over the previous 28 days [43]; in this study, the EDE-Q showed an excellent internal consistency (α = 0.961). The Patient Health Questionnaire (PHQ-9) is a nine-item self-report questionnaire used to evaluate the severity of depressive psychopathology [44]; in this study, α = 0.868. The Positive and Negative Affect Schedule (PANAS) is a 20-item self-report measure for the evaluation of positive and negative affect (e.g., enthusiasm, activity, fear, shame); items are rated on a five-point Likert scale and clustered into two subscales: PANAS-positive and PANAS-negative [45]; in this study, PANAS-positive α = 0.877, PANAS-negative α = 0.923. The need threat scale (NTS) is a 20-item questionnaire [31] usually completed after the Cyberball game that assesses feelings of distress on four dimensions: belonging (“I felt rejected”), self-esteem (“I felt liked”), meaningful existence (“I felt invisible”), and control (“I felt powerful”) on a five-point scale from “Not at all” to “Extremely”, with higher scores indicating greater distress. This scale was specifically designed to evaluate the cognitive and emotional effects of the Cyberball task [46]; in this study, α = 0.786. Two more items evaluated on a five-point Likert scale were added to the NTS to assess the extent to which participants felt excluded from the game (“I was ignored” and “I was excluded”) and were used to calculate the variable manipulation check, as suggested by the previous literature [47]. The participants were also asked to estimate the percentage of ball tosses they received by marking a point on a 10-cm long visual analogue scale. Finally, they evaluated their own levels of perceived stress (i.e., stress-related scale: SRS) and thoughts about binging or restricting (i.e., thoughts about binge scale: TBS; thoughts about restrict scale: TRS) using a seven-point Likert scale.

#### 2.2.2. Cyberball Task

The Cyberball task is a computerized ball-toss game used to evaluate the psychological effects of exclusion and inclusion in a virtual social situation [31]. Before the task, participants were told they would be playing a virtual ball-toss game with two unknown female participants who were online with them. The software is free—https://www.empirisoft.com/cyberball.aspx (accessed on 1 February 2023)—and the game is performed with a laptop, on which the participants see a graphical representation of each of the other two players near a corner of their screens. The game consisted of either an exclusion or overinclusion block of 30 ball tosses each; participants were randomly allocated to play one block or the other [41,48]. In the exclusion condition, participants received the ball four times (13% of the tosses). In the overinclusion condition, participants received the ball almost every time the computer tossed the ball (46% of the tosses). The ball receiving rates were decided based on previous literature on various psychiatric conditions, where overinclusion was rated as a specific social trigger for participants. In contrast, average inclusion might not be sufficient due to the possible tendency to expect an exclusion in ED population, or the negative interpretation of positive evaluations reported in the previous literature [9,41,49,50].

### 2.3. Procedure

**Figure 1:**
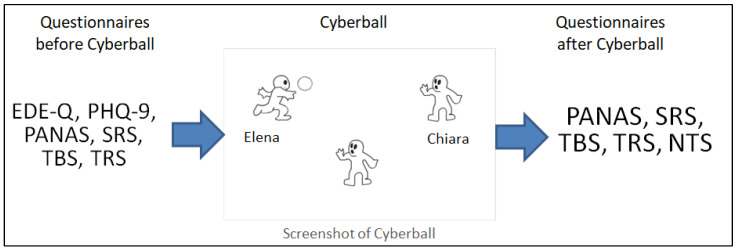
Graphical representation of the procedure. Before the task, participants filled out a series of questionnaires that were partially repeated after the task to record their emotions and thought changes. EDE-Q: eating disorder examination questionnaire; PHQ-9: Patient Health Questionnaire; PANAS: Positive and Negative Affect Schedule; SRS: stress-related scale; TBS: thoughts about binge scale; TRS: thoughts about restrict scale; NTS: need threat scale.

The study used a single-session design. After signing the consent form, participants completed the self-report questionnaires (EDE-Q, PHQ-9, PANAS, SRS, TBS, and TRS) and were randomly assigned to play either the exclusion or overinclusion block of the Cyberball game. The game was always played in the afternoon and in the same room, using a 17-inch laptop. After playing the game, participants completed the NTS, rated the percentage of ball tosses received, and completed the post-test self-report questionnaires (PANAS, SRS, TBS, and TRS). Finally, participants were debriefed according to the international guidelines regarding deception [51]. See Figure 1 for a graphical representation of the procedure. 

### 2.4. Statistical Analyses

Different analyses of variances (ANOVAs) with Bonferroni-corrected post hoc tests were calculated to examine group differences in sample characteristics, evaluating differences between clinical and non-clinical subgroups separately for each experimental block. A comparison between the participants in the excluded and overincluded conditions was performed for the demographic variables using a *t* test for independent samples. As suggested by the literature [33], a specific variable called manipulation check was created using two specific items included in the NTS scale to explicitly evaluate the participants’ perception of being manipulated during the task. The difference between groups playing the exclusion and overinclusion blocks was estimated using a *t* test for independent samples. The pre-and-post Cyberball PANAS, SRS, TBS, and TRS scores were tested with a series of general linear models (GLMs) for repeated measures with Tukey HSD (honestly significant difference) post hoc analysis. A first analysis level was characterized by comparing the two conditions separately, then a series of GLMs with two factors between groups (condition and diagnosis) and one factor within group (time) was performed. Different *t* tests for paired samples were applied for the evaluation of the effect of the Cyberball task and are reported in the Supplementary Materials (Tables S1–S4). Different correlation analyses were performed for each subgroup using Pearson’s correlations for the subscales of the NTS and the differences (D, calculated as pre- minus post- scores) in PANAS, SRS, TBS, and TRS scales. The α was corrected for multiple comparisons with the Bonferroni correction, with *p* values equal to or lower than 0.013 considered significant, with the exception of the GLM analysis, which was subject to an HSD correction as specified above. The effect sizes were calculated with partial eta squared (ηp^2^). Cronbach’s α was measured to evaluate the internal consistency of the questionnaires. All data were analyzed using IBM SPSS Statistics 25.0 software (SPSS, Chicago, IL, USA).

## 3. Results

The demographic and psychological characteristics of the participants are summarized in Table 1. For the age of participants, underage participants comprised seven with AN (17%), five with BN (13%), three with BED (13%), and five in the HW group (10%), with no significant differences in distribution (*p* = 0.201). Nineteen patients (16%) were being medicated with selective serotonin reuptake inhibitors or benzodiazepines at the time of testing: 10 in the overinclusive condition (four patients with AN, three with BN, and three with BED) and nine in the excluded one (three patients with AN, three with BN, and three with BED). The main results were confirmed after the exclusion of these participants.

No significant differences emerged between the participants in the excluded and included conditions regarding the main demographic and psychological characteristics considered at baseline; for more information, see Supplementary Materials.

### 3.1. Exclusion Condition

Means, standard deviations, and the results of the post hoc tests are reported in Table 2, while the following paragraph reports the results of the between group comparisons.

**Figure 2:**
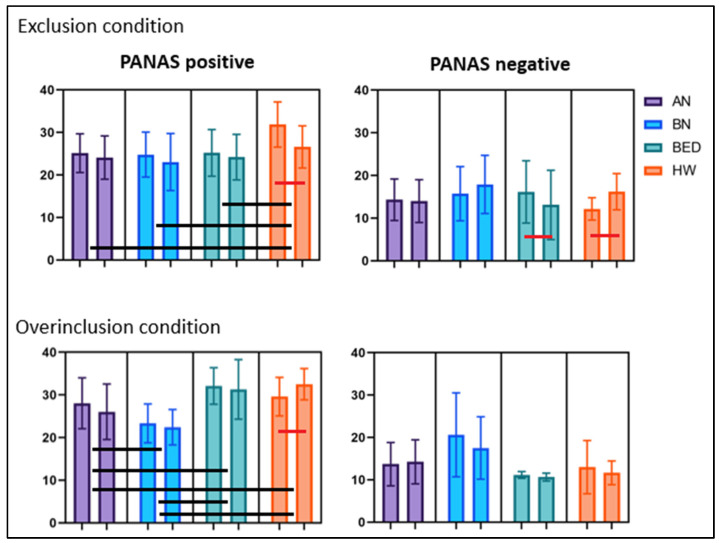
Graphical representation of the PANAS scores, with means and standard deviations. For all the pairs of bars, the one on the left is pre-Cyberball, and the one on the right is post-Cyberball. The bars show the presence of a significant difference between pre and post comparisons (red bars) or between diagnoses (black bars).

**Figure 3:**
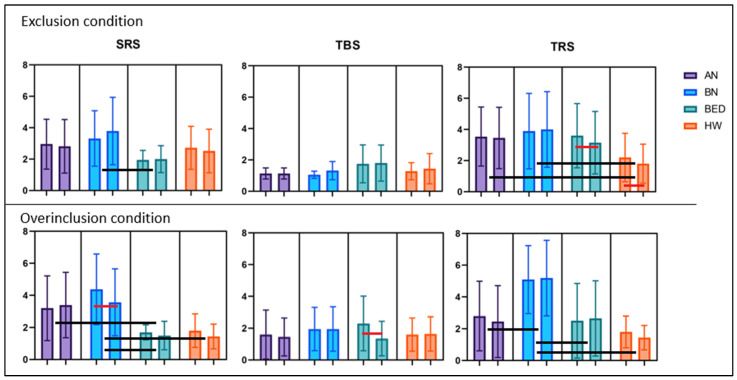
Graphical representation of the SRS, TBS, and TRS in the Cyberball conditions, with means and standard deviations. For all the pairs of bars, the one on the left is pre-Cyberball, and the one on the right is post-Cyberball. The bars show the presence of a significant difference between pre and post (red bars) comparisons or between diagnoses (black bars).

Participants with AN showed lower scores for the NTS subscales than HW, BED, and BN participants (see Table 2). The evaluation of the positive subscale of the PANAS revealed the presence of significant main effects of time (F(1, 79) = 26.810, *p* < 0.001) and diagnosis (F(3, 79) = 5.169, *p* = 0.003), as well as a significant time by diagnosis interaction (F(3, 79) = 6.225, *p* = 0.001). Post hoc analyses showed that HW reported higher scores than all of the ED groups and was the only group that displayed a significant decrease in positive emotions following the Cyberball task (*t*(24) = 7.582, *p* < 0.001; see Supplementary Materials for the pre and post Cyberball comparisons). A significant time by diagnosis interaction was also observed for the negative subscale of the PANAS (F(3, 79) = 11.328, *p* < 0.001) with HW displaying an increase in negative emotions (*t*(24) = −5.630, *p* < 0.001) and BED patients displaying a significant decrease in negative emotions (*t*(19) = 2.977, *p* = 0.008) following the exclusion condition. Regarding the SRS scale, a significant main effect of diagnosis by time emerged (F(3, 82) = 4.467, *p* = 0.006), with BN patients displaying the highest level of stress and BED patients the lowest. No differences due to exposure to the Cyberball task, however, were observed in any of the groups. In the TBS scale, no significant effect of diagnosis by time emerged in the change of the thoughts comparing before and after the Cyberball task (F(3, 82) = 5.223, *p* = 0.025). Lastly, in the TRS scale the analyses revealed the presence of significant main effects of time (F(1, 82) = 7.167, *p* = 0.009), and diagnosis (F(3, 82) = 4.193, *p* = 0.008), along with a significant time by diagnosis interaction (F(3, 82) = 3.619, *p* = 0.013), with both HW and BED patients displaying a significant reduction in thoughts about restriction (BED: *t*(19) = 2.651, *p* = 0.016; HW: *t*(24) = 2.619, *p* = 0.015). Post hoc tests revealed that both BN and AN patients reported significantly higher thoughts about restricting eating than HW. See Figure 2 and Figure 3 for a graphical representation of the data.

### 3.2. Overinclusion Condition

Means, standard deviations, and the results of the post hoc tests are reported in Table 2, while the following paragraph reports the results of the between group comparisons.

The overinclusion block demonstrated the following effects: BN patients showed lower scores regarding fundamental psychological needs than both BED patients and HW, and patients with AN and BED reported a higher rate of estimated passes than patients with BN and HW (see Table 2). The analyses conducted on the positive subscale of the PANAS revealed a significant main effect of diagnosis (F(3, 80) = 14.563, *p* < 0.001) and time by diagnosis interaction (F(3, 80) = 8.860, *p* < 0.001). Post hoc tests showed that patients with BN displayed the lowest score, followed by patients with AN, and finally by patients with BED and HW, who had the same score. With regards to the interaction, while HW displayed a significant increase in positive emotions following the overinclusion condition (*t*(24) = −3.565, *p* = 0.002), patients with AN displayed a decrease (*t*(19) = 2.629, *p* = 0.015; not significant for this study). In the negative subscale of the PANAS, the analyses revealed a significant main effect of time (F(1, 80) = 8.451, *p* = 0.005), and diagnosis (F(3, 80) = 9.460, *p* < 0.001) but only a tendency to significance for time by diagnosis interaction, considering the corrected *p* value (F(3, 80) = 3.580, *p* = 0.017). The analyses conducted on the SRS scale revealed a main effect of time (F(1, 82) = 6.554, *p* = 0.012), a main effect of diagnosis (F(3, 82) = 8.500, *p* < 0.001), and a significant time by diagnosis interaction (F(3, 82) = 3.423, *p* = 0.021). Post hoc testing showed that patients with BN and AN reported a higher stress level than BED patients, and only patients with BN displayed a significant reduction in the reported stress level (*t*(20) = 3.782, *p* = 0.001). Regarding the TBS scale, a significant main effect of time (F(1, 82) = 28.654, *p* < 0.001) and a significant time by diagnosis interaction (F(3, 82) = 14.780, *p* < 0.001) were found with patients with BED displaying a significant decrease in thoughts about binging (*t*(19) = 4.819, *p* < 0.001). Lastly, the analyses conducted on TRS showed only a main effect of diagnosis (F(3, 82) = 14.307, *p* < 0.001), with BN patients displaying higher scores on this scale. No differences due to exposure to the Cyberball task, however, were observed in any of the groups. See Figure 2 and Figure 3 for a graphical representation.

### 3.3. Conditions Comparison

Looking at the effects between exclusion and overinclusion conditions, we found significant differences for all the NTS subscales and the percentage of the ball passes, with participants who reported higher scores after overinclusion condition (*p* < 0.001 for all the subscales—belonging, self-esteem, meaningful existence, control, and manipulation check—and *p* = 0.003 for the percentage of the ball passes). Looking at the interaction between conditions and groups, we found significant differences for belonging (F(3, 164) = 4.308, *p* = 0.006, ηp^2^ = 0.860), self-esteem (F(3, 164) = 3.896, *p* = 0.010, ηp^2^ = 0.820), meaningful existence (F(3, 164) = 4.800, *p* = 0.003, ηp^2^ = 0.897), the percentage of the ball passes (F(3, 164) = 9.252, *p* < 0.001, ηp^2^ = 0.996), and a tendency to being significant for the control (F(3, 164) = 3.109, *p* = 0.028, ηp^2^ = 0.717), but no differences in regard to the manipulation check (F(3, 164) = 0.502, *p* = 0.681, ηp^2^ = 0.151). Looking at pairwise comparisons, significant differences emerged between BN and BED (*p* = 0.001), and BED and HW (*p* = 0.037), for the percentage of the ball passes. For belonging, significant differences emerged between AN and BED (*p* = 0.001), AN and HW (*p* = 0.022), and BN and BED (*p* = 0.009). For self-esteem, significant differences emerged between AN and BED (*p* = 0.001), AN and HW (*p* = 0.016), BN and BED (*p* < 0.001), and BN and HW (*p* < 0.001). Finally, for meaningful existence, significant differences emerged between AN and BED (*p* = 0.001), AN and HW (*p* < 0.001), and BN and HW (*p* = 0.027).

Looking at the interactions of time, conditions, and diagnoses, we found several significant main effects. The PANAS positive subscale showed a significant interaction (F(3, 159) = 14.686, *p* < 0.001), with significant comparisons between diagnoses at the post hoc analysis: AN ≠ HW with *p* < 0.001, BN ≠ BED with *p* < 0.001, and BN ≠ HW with *p* < 0.001. The same was for the PANAS negative subscale, which showed a significant interaction (F(3, 159) = 11.331, *p* < 0.001) with specific differences between diagnoses: AN ≠ BN with *p* = 0.009, BN ≠ BED with *p* < 0.001, and BN ≠ HW with *p* < 0.001. Looking at the SRS scale, we found a significant interaction (F(3, 164) = 3.444, *p* = 0.013) with specific differences between diagnoses: AN ≠ BED with *p* = 0.001, BN ≠ BED with *p* < 0.001, and BN ≠ HW with *p* = 0.002. The TBS scales also showed significant interaction (F(3, 164) = 10.740, *p* < 0.001) with only one significant difference at the post hoc analysis: AN ≠ BED with *p* = 0.036. Differently, no significant interaction was found for the TRS scale (F(3, 164) = 1.411, *p* = 0.241).

### 3.4. Correlations

In the excluded condition, correlation analyses found that while both HW and AN patients showed no significant results among the included psychological variables; the BN and BED groups showed negative correlations between fundamental needs (NTS scores) evaluated after the Cyberball task and the modification of their emotional states. Moreover, BN patients showed a negative correlation between the meaningful existence subscale and the change in their stress level (*r* = −0.696), while BED patients showed a negative correlation between the belonging NTS subscale and changes in their thoughts about restriction level (*r* = −0.728). In the overinclusion condition, BN patients showed negative correlations between changes in all of the thoughts scores and part of the NTS scores (meaningful existence and DSRS, *r* = −0.606; belonging and DTBS, *r* = −0.690; control and DTBS, *r* = −0.564; self-esteem and DTRS, *r* = −0.610), while BED patients showed positive correlations (belonging and DSRS *r* = 0.612; self-esteem and DSRS, *r* = 0.757; meaningful existence and DSRS, *r* = 0.734; control and DSRS, *r* = 0.717; control and DTBS, *r* = 0.604; self-esteem and DTRS, *r* = 0.738). No correlations emerged for the HW and AN groups. See Supplementary Materials for details. Regarding age at menarche, no significant correlation emerged in either condition or any of the groups.

## 4. Discussion

The current study examined changes in emotions, stress, and eating disorder attitudes after a simulated social overinclusion or exclusion scenario via a ball-tossing game. This is the first study that examined the effect of social exclusion and overinclusion across the ED spectrum and aimed to extend the previous findings in patients with AN, looking for similarities or differences between the entire spectrum of patients with ED.

Evaluation of the perception of ball passes showed that all the subgroups could discriminate between exclusion and overinclusion in the ball-toss game; our results confirmed preliminary evidence about the ability of AN patients to determine the nature of a social scenario [1,41] and added new data for patients with BN and BED. For participants’ perception of their overinclusion or exclusion in the game, more significant differences emerged during the overincluding paradigm, confirming that social comparison is a vulnerability aspect that should be carefully considered when treating ED patients [52].

Some peculiarities concerning the specific ED diagnosis also emerged. Patients with AN demonstrated psychological difficulty in being excluded by peers with lower scores at the evaluation after the Cyberball task [41], while BN patients showed that overinclusion by peers could be more complex to manage.

Indeed, patients with BN reported that interpersonal exposure induced by the over including condition results in lower scores for self-esteem and sense of belonging than other participants. Both of these elements agree with the interpersonal model for ED [15], which shows that AN patients have more fear of negative evaluations, while BN patients have higher rates of negative interactions with others. Our results align with the impaired social effects of interpersonal interaction that are linked, in BN patients, more to the reduction of negative emotions than to an increase in positive ones (see above). Our results highlighted different risk factors in the interpersonal domain for each clinical group, demonstrating specific psychological needs that should also be considered in the therapeutic process [53], especially looking at personalized approaches [54].

In line with our previous study, the data indicate difficulties in emotional reactivity in AN patients after being ostracized [41], reporting a higher negative impact on self-esteem, sense of belonging, and psychological well-being. The new data, however, show that this aspect is a key feature characterizing the entire ED spectrum, with no differences among ED diagnoses (AN, BN, BED). The difficulties in understanding their emotional states may suggest a possible cognitive factor that has a role in developing and maintaining dysfunctional behaviors across the entire spectrum [52,55]. Indeed, looking at the affective response to the Cyberball paradigm, no significant differences emerged between clinical groups in the excluded condition. Moreover, significant changes in the emotional state were reported only by healthy individuals. This aspect might underline an impairment in ED patients’ ability to understand their emotional states linked to interpersonal interactions, in line with dysfunctional emotional reactiveness and interpersonal models of EDs [56]. Patients might present a reduced emotional reactivity to this kind of social interaction or task, corroborating the previous data about the possible presence of impaired emotional reactivity in patients with an ED [57]. Indeed, different studies have pointed out the possible role of emotion regulation in EDs, showing the relevance of targeting emotional skills in treatments [58,59]. However, another possible explanation is the presence of high alexithymia, a severe trait present in patients with EDs, or dissociation during the task due to a non-acceptance of emotional changes [60,61].

Interestingly, patients with BED reduced their negative emotional state after being excluded, which was linked to the high level of belonging reported on the NTS scale. The same results have been found in post-bariatric patients [62], where the same correlations were found, showing a similarity in interpersonal skills difficulties [63] that might be explained by a shared impaired capacity to understand and code their own emotions [21]. Indeed, although the link between precipitating negative affect and binging is already well documented, little is known about the cognitive or behavioral chain; moreover, recent evidence has demonstrated the importance of intra-individual variability in the precipitating affective processes and the control of food-related impulsivity [64]. Another possible explanation might be the presence of an emotional detachment response to acute social exclusion that has been proposed as an “invisible shield”, with an automatic emotion process in which positive emotions become highly accessible [65]. This process might be true for people with high social negative evaluations and exclusion [23,66], such as individuals with BED [67]. For these reasons, future studies might focus on emotional processing in individuals with BED, and could evaluate the implementation of emotional recognition and interpretation in a psychological approach that has already shown interesting effects in the reduction of food impulsivity [68,69], looking at personalized and improved treatment approaches [70].

In the overincluding paradigm, patients with BN showed a significant decrease in negative emotions, with a possible positive effect of social interactions on negative emotions but not on positive emotions that may be linked to an impaired reward from social interactions [71]. Interestingly, even if overinclusion reduced stress levels in BN patients, no differences emerged regarding eating thoughts, corroborating the evidence that patients with BN could present a dissociation between emotional states and psychopathological behavioral responses [28].

Several studies in the literature have pointed out the role of the age of menarche in emotional regulation and social cognition [72,73,74]. Our data do not support this aspect, but the heterogeneity of the sample and the specificity of the relationship between EDs and menarche might be considered for future evaluations. Indeed, bodily changes after menarche are possible stressors for developing an ED, impacting the physiological relationships between the hormonal cycle and the development of cognitive functions such as emotional regulation due to dysfunctional eating behaviors [75,76,77].

No relationships emerged between psychological need, psychopathology, and eating thoughts in the control group, which differed from previous literature and may be linked to the different tasks applied [35]. Overall, the HW group reported the largest modulation of emotional states in this experiment, corroborating the idea that individuals with EDs apply maladaptive strategies in interpersonal emotion regulation [19]. These data are in line with the possible role of rumination and thought suppression as cognitive dysfunctional emotion regulation strategies in individuals with ED [78]. Rumination—an obsessive and passive focus on the meaning, causes, and consequences of emotions—and suppression thereof—disengagement from unwanted thoughts—are maladaptive strategies to disengage from the context. They have been related to dysfunctional eating behaviors in patients, with a paradoxical increase in negative emotions and cognitions [78] that might also be a difficult trait in patients [79].

Although the relevance of interpersonal relationships has been noted in several treatment approaches, from interpersonal therapy to family-based therapy and the Maudsley Model (MANTRA), more studies are needed [19]. Emotional regulation has been shown to have a relevant role in the treatment pathway, possibly impacting the therapeutic alliance and recovery. Our data showed a dynamic response modulation in patients with AN, suppression of emotional changes and increased eating behaviors in BN patients via social manipulation, and difficulties in emotional self-reading in BED patients linked to social cues. This indicates specific differences regarding social cognition in the ED spectrum that, as suggested by a recent literature review, require more study [1]. These aspects might also be important in different scenarios and have been pointed out as crucial, for example, during the COVID-19 pandemic [80]. Indeed, the disruption of social connections and routines has been related to more severe psychopathology, and impaired coping strategies in social situations might explain different responses to the pandemic [81,82,83], calling for new studies in the field.

This is the first study to apply the Cyberball task to the whole spectrum of EDs, and the main strength is the experimental psychopathological approach applied, but upon examining our results, several limitations should be considered. First, the self-reporting nature of the data could affect the self-report results, and future studies should include different ways to record participants’ responses (e.g., skin conductance, heartbeat). Second, in seeking increasing homogeneity, we included only female participants, but this choice could limit the generalizability of the results. Third, this is a laboratory-based experiment. Even if none of the participants were familiar with the Cyberball task before the session or reported doubts about the real presence of the other participants, more studies are needed to confirm our results and to compare a laboratory task to real-world situations. Fourth, our results need to be replicated and confirmed with larger populations and from different experimental studies. Finally, in evaluating the psychological effects of the Cyberball task, we included participants of different ages, but we may have lost some specific differences between adolescents and adults that could be investigated in future studies.

## 5. Conclusions

Despite some limitations, this is the first study to evaluate the effect of social exclusion and overinclusion across the ED spectrum. Our results highlight the relevant role of the interpersonal domain in EDs, showing possible trigger mechanisms in either overinclusion or exclusion that could directly affect eating behaviors. Indeed, participants from the general population shown the largest emotional modulation considering both conditions, while patients with AN showed a different vulnerability toward being ostracized and overincluded than those with BN or BED, suggesting that more studies are needed in order to comprehend patients’ ability to understand social, emotional, and interpersonal scenarios [1,20]. Interpersonal emotion regulation and its relationship with eating behaviors in patients with EDs should be evaluated with different laboratory tasks, examining possible differences due to the duration of the disorder or age of the participants (adolescence vs. adulthood). Future studies should include different psychological constructs such as social anxiety and fear of positive/negative evaluations, seeking improved differentiations that could help clinicians create personalized interventions. Indeed, the research should look for specific training that could improve the coping strategies and treatment outcomes proposed in the literature, from the cognitive approach to emotional cues to approaches more linked to the third generation of cognitive therapies [84,85].

## Figures and Tables

**Table 1:** Demographic and clinical characteristics of the participants.

	AN *n* = 42	BN *n* = 40	BED *n* = 40	HW *n* = 50	F	*p* ηp^2^	Post Hoc
Age, years	24.62 (8.26)	23.60 (6.86)	30.28 (9.67)	24.16 (3.41)	7.454	**<0.001** 0.117	AN < BED (*p* = 0.003) BN < BED (*p* < 0.001) HW < BED (*p* = 0.001)
BMI, kg/m^2^	16.68 (0.87)	20.05 (2.06)	33.13 (10.05)	21.45 (2.96)	75.419	**<0.001** 0.574	AN < BN (*p* = 0.007) AN < BED (*p* < 0.001) AN < HW (*p* < 0.001) BN < BED (*p* < 0.001) HW < BED (*p* < 0.001)
Menarche, years	12.71 (1.29)	11.98 (1.19)	11.79 (1.27)	12.38 (1.58)	3.887	**0.010** 0.065	BED < AN (*p* = 0.014)
Education, years	13.98 (2.79)	15.21 (2.95)	14.83 (3.09)	16.06 (2.74)	4.106	**0.008** 0.069	AN < HW (*p* = 0.004)
Illness duration, months	5.83 (5.99)	5.76 (6.72)	12.14 (9.53)	-	22.638	**<0.001** 0.307	AN < BED (*p* < 0.001) BN < BED (*p* < 0.001)
PHQ-9	10.02 (4.57)	12.70 (5.75)	10.65 (4.29)	6.14 (3.94)	15.975	**<0.001** 0.222	HW < AN (*p* = 0.001) HW < BN (*p* < 0.001) HW < BED (*p* < 0.001)
EDE-Q Global	13.65 (8.23)	20.21 (8.59)	20.01 (5.36)	7.36 (6.44)	32.269	**<0.001** 0.366	HW < AN (*p* < 0.001) AN < BN (*p* < 0.001) AN < BED (*p* = 0.001) HW < BN (*p* < 0.001) HW < BED (*p* < 0.001)

Table reports means and (standard deviation), with ANOVAs’ F and post hoc analyses. BMI: body mass index; AN: anorexia nervosa; BN: bulimia nervosa; BED: binge eating disorder; HW: healthy women; PHQ-9: patient health questionnaire; EDE-Q: eating disorder examination questionnaire; post hoc analysis with Bonferroni correction. Significant *p* values after Bonferroni correction are reported in bold.

**Table 2:** Need threat scale (NTS) results and comparisons.

**Exclusion Condition**							
	**AN** ***n* = 22**	**BN** ***n* = 19**	**BED** ***n* = 20**	**HW** ***n* = 25**	**F**	***p*** **ηp^2^**	**Post Hoc**
% passes	16.70 (8.30)	15.30 (8.30)	17.10 (8.90)	21.20 (7.80)	2.181	0.096 0.074	
Belonging	9.95 (3.61)	12.53 (4.51)	13.30 (2.03)	12.48 (3.11)	3.934	**0.011** 0.126	AN < BED (*p* = **0.012**)
Self-esteem	10.50 (3.79)	11.95 (5.17)	14.20 (2.14)	14.00 (3.33)	4.922	**0.003** 0.153	AN < BED (*p* = **0.011**) AN < HW (*p* = **0.011**)
Meaningful existence	10.00 (4.27)	13.42 (4.68)	13.00 (2.43)	14.32 (3.20)	5.728	**0.001** 0.173	AN < BN (*p* = 0.026) AN < HW (*p* = **0.001**)
Control	7.68 (1.81)	8.84 (3.53)	8.85 (2.39)	9.52 (2.43)	2.024	0.117 0.069	
Manipulation check	7.41 (2.13)	6.37 (2.29)	6.90 (2.38)	6.52 (2.31)	0.890	0.450 0.032	
**Overinclusion Condition**							
	**AN** ***n* = 20**	**BN** ***n* = 21**	**BED** ***n* = 20**	**HW** ***n* = 25**	**F**	***p*** **ηp^2^**	**Post Hoc**
% passes	67.50 (15.80)	53.90 (13.50)	72.00 (5.20)	55.80 (12.20)	10.828	**<0.001** 0.284	BN < AN (*p* = **0.004**) HW < AN (*p* = 0.013) BN < BED (*p* < **0.001**) HW< BED (*p* < **0.001**)
Belonging	20.25 (3.31)	17.81 (3.12)	21.95 (2.74)	21.16 (3.05)	7.221	**<0.001** 0.209	BN < BED (*p* < **0.001**) BN < HW (*p* = **0.002**)
Self-esteem	16.70 (4.85)	12.71 (3.86)	19.05 (2.52)	17.60 (3.49)	10.909	**<0.001** 0.285	BN < AN (*p* = **0.006**) BN < BED (*p* < **0.001**) BN < HW (*p* < **0.001**)
Meaningful existence	18.80 (4.25)	17.05 (2.04)	20.90 (2.10)	20.32 (2.75)	7.462	**<0.001** 0.214	BN < BED (*p* < **0.001**) BN < HW (*p* = **0.002**)
Control	15.95 (5.96)	13.05 (3.37)	13.45 (4.12)	16.32 (3.08)	3.483	0.019 0.113	BN < HW (*p* = 0.050)
Manipulation check	2.60 (1.31)	2.29 (0.72)	2.00 (0.00)	2.12 (0.60)	2.191	0.095 0.074	

AN: anorexia nervosa; BN: bulimia nervosa; BED: binge eating disorder; HW: healthy women; post hoc analysis with Bonferroni correction. Significant *p* values after Bonferroni correction are report-ed in bold.

## Data Availability

The datasets used and analyzed during the current study are available from the corresponding author upon reasonable request.

## Supplementary Materials

The following supporting information can be downloaded at: https://www.mdpi.com/article/10.3390/nu15041021/s1. Table S1: Comparisons between participants in the excluded and overincluded conditions at baseline; Table S2: Correlation analyses exclusion condition; Table S3: Correlation analyses overinclusion condition; Table S4: Evaluation of change due to the Cyberball task.
