# Supplementary material for: Virtual Rejection and Overinclusion in Eating Disorders: An Experimental Investigation of the Impact on Emotions, Stress Perception, and Food Attitudes
Source: Nutrients. 2023 Feb 17;15(4):1021. doi: 10.3390/nu15041021 (PMC9965581; doi:10.3390/nu15041021)
Supplement: Supplementary file 1 [file nutrients-15-01021-s001.zip › nutrients-2227252-supplementary.pdf]

Virtual Rejection and Overinclusion in Eating Disorders: An Experimental Investigation of the Impact on Emotions, Stress Perception, and Food Attitudes

Paolo Meneguzzo [1,2,*], Valentina Meregalli [1,2], Enrico Collantoni [1,2], Valentina Cardi [3,4], Elena Tenconi [1,2] and Angela Favaro [1,2]

1 Department of Neuroscience, University of Padova, Via Giustiniani 2, 35128 Padova, Italy
2 Neuroscience Padova Center, University of Padova, Via Orus 2, 35128 Padova, Italy
3 Department of Psychological Medicine, Institute of Psychiatry, Psychology and Neuroscience, King's College London, London WC2R 2LS, UK
4 Department of General Psychology, University of Padova, Via Venezia 8, 35128 Padova, Italy
* Correspondence: paolo.meneguzzo@unipd.it; Tel: +39-0498213830

Supplementary Materials

Table S1. Comparisons between participants in the excluded and overincluded conditions at baseline.

	**Excluded Condition**	**Included Condition**	***t*** ***p***
Age, years	25.65 (7.58)	25.48 (7.77)	0.149 0.882
BMI, kg/m^2	21.57 (4.76)	23.85 (9.62)	−1.968 0.061
Menarche, years	12.10 (1.37)	12.35 (1.39)	−1.202 0.231
Education, years	15.52 (2.76)	14.65 (3.10)	1.955 0.062
Illness duration, months	5.61 (7.62)	5.77 (7.67)	−0.128 0.899
PHQ-9	9.94 (5.34)	9.38 (4.90)	0.700 0.558
EDE-Q Global	15.56 (8.53)	14.08 (9.45)	1.073 0.285
PANAS pos pre	26.94 (5.98)	28.08 (5.95)	−1.228 0.221
PANAS neg pre	14.52 (5.64)	14.40 (6.85)	0.134 0.894
SRS pre	2.73 (1.45)	3.02 (1.88)	−1.133 0.259
TBS pre	1.49 (0.72)	1.95 (1.52)	−2.155 0.130
TRS pre	3.23 (2.08)	3.00 (2.30)	0.696 0.487

Means and standard deviations are reported.

Table S2. Correlation analyses exclusion condition.

	D$_{PANAS-pos}$	D$_{PANAS-neg}$	D$_{TBS}$	D$_{TRS}$	D$_{SRS}$
AN					
Belonging	0.256	−0.124	-	−0.148	−0.321
Self-esteem	0.428	−0.395	-	−0.021	−0.078
ME	0.562	−0.349	-	−0.282	−0.361
Control	0.177	0.274	-	−0.149	−0.366
BN					
Belonging	0.074	0.227	−0.101	0.061	−0.699*
Self-esteem	0.346	0.346	0.196	−0.044	−0.375
ME	−0.755*	−0.623*	0.040	−0.160	−0.696*
Control	−0.687*	0.177	−0.006	−0.012	−0.420
BED					
Belonging	−0.385	−0.431	−0.385	−0.728*	−0.597*
Self-esteem	−0.543	−0.763*	−0.543	0.139	−0.395
ME	−0.606*	−0.277	−0.314	−0.214	−0.214
Control	0.347	−0.865*	−0.273	0.337	0.393
HW					
Belonging	0.066	−0.143	−0.149	0.330	0.015
Self-esteem	−0.042	−0.468	−0.313	0.229	0.165
ME	0.154	−0.353	−0.575	0.379	0.046
Control	−0.065	0.164	−0.237	0.184	0.141

AN: anorexia nervosa; BN: bulimia nervosa; BED: binge eating disorder; HW: healthy women; ME: meaningful existence; PANAS: positive and negative affect schedule; D: difference; pos: positive; neg: negative; SRS: stress related scale; TBS: thoughts about binge scale; TRS: thoughts about restraint scale. *: $p < 0.01$, only correlation with $p < 0.01$ should be considered due to the Bonferroni correction. Differences were evaluated as post-pre task for all the variables. NTS subscales (belonging, self-esteem, ME and Control) were collected after the task, while other variables were collected both pre and post.

Table S3. Correlation analyses overinclusion condition.

	D$_{PANAS-pos}$	D$_{PANAS-neg}$	D$_{TBS}$	D$_{TRS}$	D$_{SRS}$
		AN			
Belonging	0.239	−0.101	−0.227	0.003	−0.228
Self-esteem	0.391	−0.250	−0.078	0.163	−0.364
ME	0.127	0.167	0.231	0.518	−0.053
Control	0.307	0.265	0.067	0.057	0.391
		BN			
Belonging	0.107	−0.384	−0.690*	−0.298	−0.526
Self-esteem	0.486	0.563*	−0.299	−0.610*	−0.170
ME	0.412	−0.430	−0.462	−0.239	−0.606*
Control	0.572*	−0.489	−0.564*	−0.121	−0.239
		BED			
Belonging	−0.841*	−0.137	0.236	0.583	0.612*
Self-esteem	−0.385	−0.655*	0.305	0.738*	0.757*
ME	0.121	−0.439	0.571	0.511	0.734*
Control	0.213	0.681*	0.604*	0.113	0.717*
		HW			
Belonging	0.010	0.110	0.053	0.029	0.029

Self-esteem	−0.008	−0.132	0.002	0.024	−0.008
ME	−0.076	0.075	0.124	−0.020	−0.076
Control	−0.244	0.114	0.080	−0.059	−0.244

AN: anorexia nervosa; BN: bulimia nervosa; BED: binge eating disorder; HC: healthy women; ME: meaningful existence; PANAS: positive and negative affect schedule; D: difference; pos: positive; neg: negative; SRS: stress related scale; TBS: thoughts about binge scale; TRS: thoughts about restraint scale. *: $p < 0.01$, only correlation with $p < 0.01$ should be considered due to the Bonferroni correction. Differences were evaluated as pre minus post task for all the variables. NTS subscales (belonging, self-esteem, ME and Control) were collected after the task, while other variables were collected both pre and post.

Table S4. Evaluation of change due to the Cyberball task.

	**Exclusion Condition**		**Overinclusion Condition**	
	t	***p***	***t***	***p***
		AN		
PANAS positive	1.047	0.307	2.629	0.015
PANAS negative	0.381	0.707	−1.453	0.163
SRS	0.548	0.589	−0.809	0.428
TBS	1.000	0.333	0.825	0.419
TRS	1.000	0.329	1.926	0.069
		BN		
PANAS positive	1.332	0.203	1.370	0.187
PANAS negative	−1.624	0.125	4.656	< 0.001
SRS	−1.094	0.255	3.782	0.001
TBS	−2.041	0.033	1.746	0.096
TRS	−1.000	0.331	−0.698	0.493
		BED		
PANAS positive	2.304	0.033	0.986	0.336
PANAS negative	2.977	0.008	2.342	0.030
SRS	−0.370	0.716	1.073	0.297
TBS	−1.453	0.163	4.819	<0.001
TRS	2.651	0.012	−0.339	0.739
		HW		
PANAS positive	7.582	<0.001	−3.565	0.002
PANAS negative	−5.630	<0.001	1.209	0.239
SRS	0.775	0.446	1.445	0.161
TBS	−1.000	0.327	1.000	0.327
TRS	2.619	0.012	1.674	0.107

t-test for paired sample. Means and standard deviations are reported in the main text.
